# Supplementary material for: Acceptance of the Human Papillomavirus Vaccine among General Men and Men with a Same-Sex Orientation and Its Influencing Factors: A Systematic Review and Meta-Analysis
Source: Vaccines (Basel). 2023 Dec 22;12(1):16. doi: 10.3390/vaccines12010016 (PMC10819436; doi:10.3390/vaccines12010016)
Supplement: Supplementary file 1 [file vaccines-12-00016-s001.zip › vaccines-2716278-supplementary.pdf]

Supplementary Material

Acceptance of the human papillomavirus vaccine among general men and men with a same-sex orientation and its influencing factors: A systematic review and meta-analysis

Fang Shen#; Yuxia Du#; Kexin Cao; Can Chen; Mengya Yang; Rui Yan; Shigui Yang*

Department of Emergency Medicine, Second Affiliated Hospital, Department of Public Health, State Key Laboratory for Diagnosis and Treatment of Infectious Diseases. The Key Laboratory of Intelligent Preventive Medicine of Zhejiang Province, Zhejiang University School of Medicine, Hangzhou 310058, China;

#These authors contributed equal.

*Correspondence author: Prof. Shigui Yang, PhD, Email: yangshigui@zju.edu.cn; Telephone: +86 13605705640, Address: 866 Yuhangtang road, Hangzhou, China

1 Supplementary Figures and Tables

1.1 Supplementary Figures

Study	Events	Total	Proportion	95%-CI	Weight (common)	Weight (random)
group = General male						
Ferris-2008	177	536	0.330	[0.291; 0.372]	1.6%	1.5%
Daley-2010	398	494	0.806	[0.768; 0.840]	1.5%	1.5%
Hernandez-2010	233	343	0.679	[0.627; 0.728]	1.0%	1.5%
Reiter-2010	109	297	0.367	[0.312; 0.425]	0.9%	1.5%
Gilbert-2011	109	296	0.368	[0.313; 0.426]	0.9%	1.5%
Katz-2011	131	165	0.794	[0.724; 0.853]	0.5%	1.5%
Colon-Lopez-2012	10	13	0.769	[0.462; 0.950]	0.0%	1.2%
Forster-2012	217	528	0.411	[0.369; 0.454]	1.6%	1.5%
Ortashi-2013	164	356	0.461	[0.408; 0.514]	1.1%	1.5%
Belani-2014	160	178	0.899	[0.845; 0.939]	0.5%	1.5%
Gellenoncourt-2014	113	328	0.345	[0.293; 0.399]	1.0%	1.5%
Giuliani-2016	52	74	0.703	[0.585; 0.803]	0.2%	1.5%
Hunter-2016	23	116	0.198	[0.130; 0.283]	0.4%	1.5%
Napolitano-2016	556	956	0.582	[0.550; 0.613]	2.9%	1.5%
Zou-2016	85	182	0.467	[0.393; 0.542]	0.6%	1.5%
Cheung-2018	234	1004	0.233	[0.207; 0.260]	3.0%	1.5%
Stanley-2018	41	51	0.804	[0.669; 0.902]	0.2%	1.4%
Wong-2019	1366	2823	0.484	[0.465; 0.503]	8.6%	1.5%
Kuang-2019	139	300	0.463	[0.406; 0.522]	0.9%	1.5%
Li-2019	433	1156	0.375	[0.347; 0.403]	3.5%	1.5%
Wang-2020	493	1274	0.387	[0.360; 0.414]	3.9%	1.5%
Ren-2020	10	102	0.098	[0.048; 0.173]	0.3%	1.5%
Farsi-2021	251	517	0.485	[0.442; 0.530]	1.6%	1.5%
Bai-2021	653	1196	0.546	[0.517; 0.574]	3.6%	1.5%
Ran-2022	250	844	0.296	[0.266; 0.328]	2.6%	1.5%
Shi-2022	360	935	0.385	[0.354; 0.417]	2.8%	1.5%
Zhang-2022	99	421	0.235	[0.195; 0.279]	1.3%	1.5%
Zhang-2022	124	373	0.332	[0.285; 0.383]	1.1%	1.5%
Gursoy-2023	172	1646	0.104	[0.090; 0.120]	5.0%	1.5%
Lv-2023	19	25	0.760	[0.549; 0.906]	0.1%	1.3%
Al-Naggar-2012	194	350	0.554	[0.501; 0.607]	1.1%	1.5%
Cooper-2018	38	160	0.237	[0.174; 0.311]	0.5%	1.5%
Hoefer-2017	229	298	0.768	[0.716; 0.815]	0.9%	1.5%
Khurana-2015	23	148	0.155	[0.101; 0.224]	0.5%	1.5%
Oh-2010	275	496	0.554	[0.509; 0.599]	1.5%	1.5%
Gottvall-2009	194	261	0.743	[0.686; 0.795]	0.8%	1.5%
Richard-2011	67	148	0.453	[0.371; 0.537]	0.5%	1.5%
Blodt-2012	19	232	0.082	[0.050; 0.125]	0.7%	1.5%
Stephens-2016	46	51	0.902	[0.786; 0.967]	0.2%	1.4%
Common effect model		19673	0.414	[0.407; 0.421]	59.7%	--
Random effects model			0.470	[0.392; 0.549]	--	58.3%
Heterogeneity: $I^2 = 99\%$, $\tau^2 = 0.0608$, $p = 0$						
group = Sexual orientation includes male						
Ferris-2008	9	26	0.346	[0.172; 0.557]	0.1%	1.3%
Hernandez-2010	66	88	0.750	[0.646; 0.836]	0.3%	1.5%
Reiter-2010	225	306	0.735	[0.682; 0.784]	0.9%	1.5%
Gilbert-2011	185	247	0.749	[0.690; 0.802]	0.8%	1.5%
Gilbert-2011	229	312	0.734	[0.681; 0.782]	0.9%	1.5%
Wheldon-2011	64	178	0.360	[0.289; 0.435]	0.5%	1.5%
Rank-2012	690	1032	0.669	[0.639; 0.697]	3.1%	1.5%
Zou-2014	60	200	0.300	[0.237; 0.369]	0.6%	1.5%
Giuliani-2016	93	129	0.721	[0.635; 0.796]	0.4%	1.5%
Zou-2016	50	186	0.269	[0.207; 0.339]	0.6%	1.5%
Zhu-2016	310	422	0.735	[0.690; 0.776]	1.3%	1.5%
Tian-2017	343	500	0.686	[0.643; 0.726]	1.5%	1.5%
Tian-2019	386	458	0.843	[0.806; 0.875]	1.4%	1.5%
Kuang-2019	75	280	0.268	[0.217; 0.324]	0.9%	1.5%
Li-2019	32	53	0.604	[0.460; 0.735]	0.2%	1.4%
Chen-2022	802	969	0.828	[0.802; 0.851]	2.9%	1.5%
Pan-2022	736	889	0.828	[0.801; 0.852]	2.7%	1.5%
Pham-2022	2114	3342	0.633	[0.616; 0.649]	10.1%	1.5%
Ran-2022	65	68	0.956	[0.876; 0.991]	0.2%	1.5%
Zhang-2022	168	325	0.517	[0.461; 0.572]	1.0%	1.5%
Lv-2023	282	315	0.895	[0.856; 0.927]	1.0%	1.5%
He -2021	350	406	0.862	[0.825; 0.894]	1.2%	1.5%
Sadlier-2016	87	281	0.310	[0.256; 0.367]	0.9%	1.5%
Cooper-2018	2	15	0.133	[0.017; 0.405]	0.0%	1.2%
Nadarzynski-2018	1076	1295	0.831	[0.809; 0.851]	3.9%	1.5%
Ye-2021	135	234	0.577	[0.511; 0.641]	0.7%	1.5%
Gray-2013	108	542	0.199	[0.166; 0.235]	1.6%	1.5%
Thomas-2011	123	191	0.644	[0.572; 0.712]	0.6%	1.5%
Common effect model		13289	0.676	[0.668; 0.684]	40.3%	--
Random effects model			0.622	[0.529; 0.711]	--	41.7%
Heterogeneity: $I^2 = 99\%$, $\tau^2 = 0.0619$, $p = 0$						
**Common effect model**		**32962**	**0.521**	**[0.515; 0.526]**	**100.0%**	**--**
**Random effects model**			**0.534**	**[0.471; 0.597]**	**--**	**100.0%**

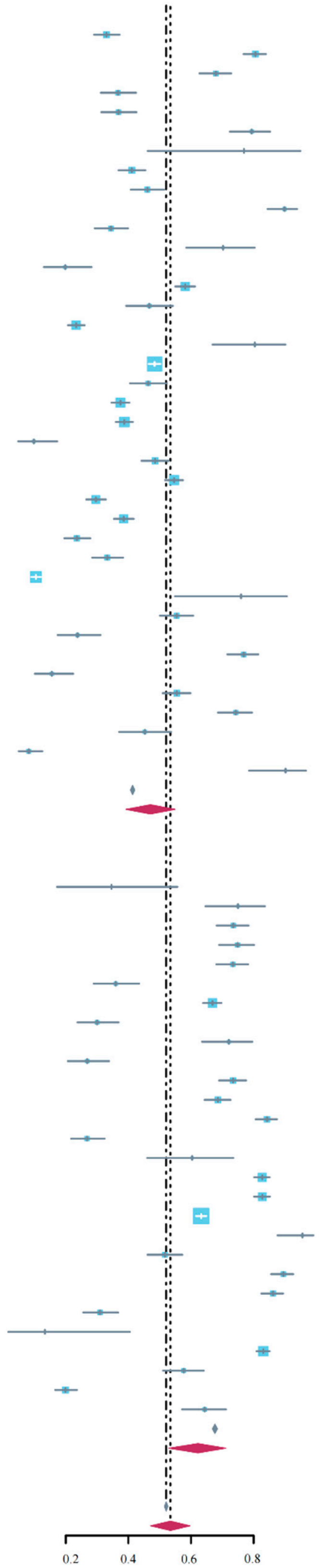

Heterogeneity: $I^2 = 99\%$, $\tau^2 = 0.0666$, $p = 0$
Test for subgroup differences (common effect): $\chi^2_1 = 2232.74$, df = 1 (p = 0)
Test for subgroup differences (random effects): $\chi^2_1 = 5.96$, df = 1 (p = 0.01)

Figure S1 Subgroup analysis of HPV vaccina acceptance rates among general men and men whose sexual orientation includes men

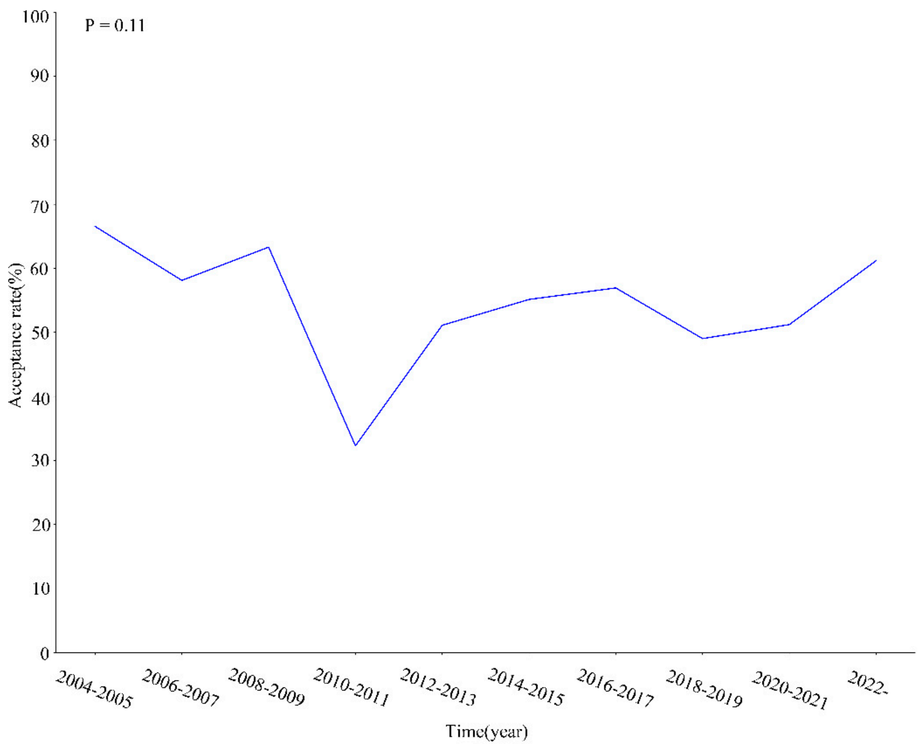

Figure S2. Changes in HPV vaccine acceptance rates over time

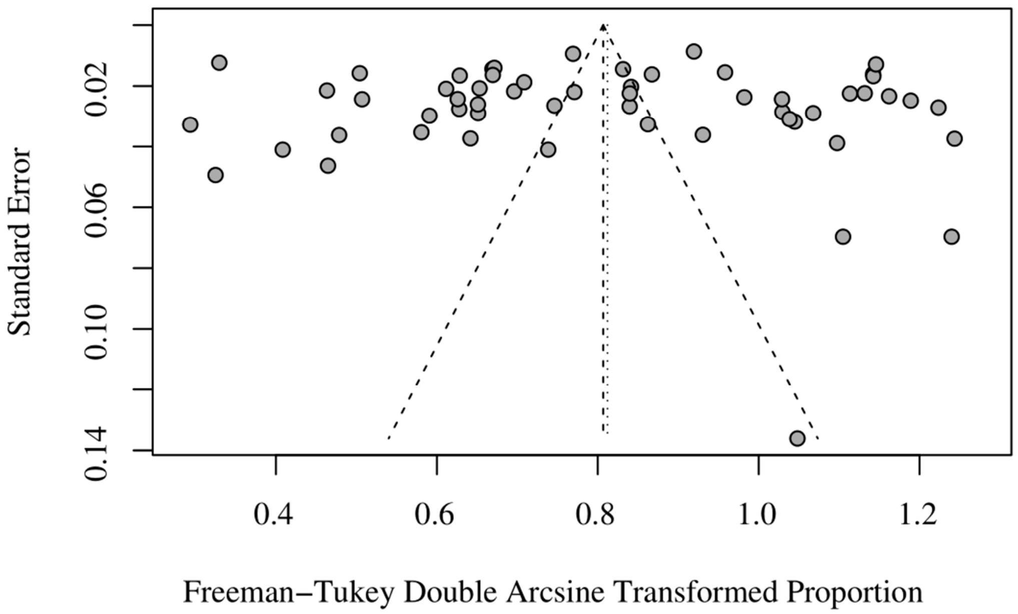

Figure S3 Funnel plot of risk bias

Study	Proportion	95%-CI	P-value	Tau2	Tau	I2
Omitting -2008	0.47	[0.39; 0.56]	.	0.0620	0.2490	99%
Omitting -2010	0.46	[0.38; 0.54]	.	0.0587	0.2423	99%
Omitting -2010	0.46	[0.39; 0.54]	.	0.0612	0.2474	99%
Omitting -2010	0.47	[0.39; 0.55]	.	0.0623	0.2495	99%
Omitting -2011	0.47	[0.39; 0.55]	.	0.0623	0.2495	99%
Omitting -2011	0.46	[0.38; 0.54]	.	0.0592	0.2432	99%
Omitting -2012	0.46	[0.39; 0.54]	.	0.0607	0.2463	99%
Omitting -2012	0.47	[0.39; 0.55]	.	0.0625	0.2499	99%
Omitting -2013	0.47	[0.39; 0.55]	.	0.0626	0.2501	99%
Omitting -2014	0.46	[0.38; 0.53]	.	0.0556	0.2359	99%
Omitting -2014	0.47	[0.39; 0.55]	.	0.0621	0.2492	99%
Omitting -2016	0.46	[0.39; 0.54]	.	0.0610	0.2469	99%
Omitting -2016	0.48	[0.40; 0.56]	.	0.0602	0.2454	99%
Omitting -2016	0.47	[0.39; 0.55]	.	0.0622	0.2494	99%
Omitting -2016	0.47	[0.39; 0.55]	.	0.0625	0.2501	99%
Omitting -2018	0.48	[0.40; 0.56]	.	0.0607	0.2465	99%
Omitting -2018	0.46	[0.38; 0.54]	.	0.0593	0.2435	99%
Omitting -2019	0.47	[0.39; 0.55]	.	0.0626	0.2501	99%
Omitting -2019	0.47	[0.39; 0.55]	.	0.0626	0.2501	99%
Omitting -2019	0.47	[0.39; 0.55]	.	0.0623	0.2496	99%
Omitting -2020	0.47	[0.39; 0.55]	.	0.0624	0.2498	99%
Omitting -2020	0.48	[0.40; 0.56]	.	0.0575	0.2398	99%
Omitting -2021	0.47	[0.39; 0.55]	.	0.0626	0.2501	99%
Omitting -2021	0.47	[0.39; 0.55]	.	0.0624	0.2498	99%
Omitting -2022	0.48	[0.40; 0.56]	.	0.0616	0.2483	99%
Omitting -2022	0.47	[0.39; 0.55]	.	0.0624	0.2497	99%
Omitting -2022	0.48	[0.40; 0.56]	.	0.0608	0.2466	99%
Omitting -2022	0.47	[0.39; 0.55]	.	0.0620	0.2490	99%
Omitting -2023	0.48	[0.40; 0.56]	.	0.0572	0.2393	98%
Omitting -2023	0.46	[0.39; 0.54]	.	0.0604	0.2458	99%
Omitting -2012	0.47	[0.39; 0.55]	.	0.0623	0.2497	99%
Omitting -2018	0.48	[0.40; 0.56]	.	0.0609	0.2467	99%
Omitting -2017	0.46	[0.38; 0.54]	.	0.0597	0.2443	99%
Omitting -2015	0.48	[0.40; 0.56]	.	0.0592	0.2433	99%
Omitting -2010	0.47	[0.39; 0.55]	.	0.0623	0.2497	99%
Omitting -2009	0.46	[0.38; 0.54]	.	0.0602	0.2453	99%
Omitting -2011	0.47	[0.39; 0.55]	.	0.0625	0.2501	99%
Omitting -2012	0.48	[0.41; 0.56]	.	0.0565	0.2376	99%
Omitting -2016	0.46	[0.38; 0.54]	.	0.0564	0.2375	99%
**Random effects model**	**0.47**	**[0.39; 0.55]**	**.**	**0.0608**	**0.2466**	**99%**

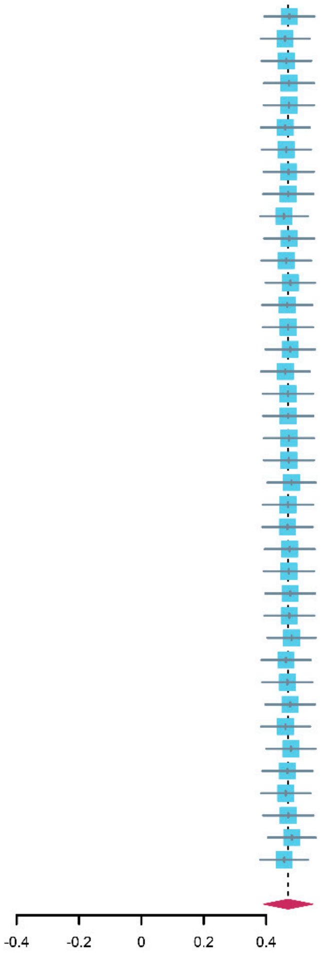

Figure S4 Forest plot for sensitivity analysis

1.2 Supplementary Tables

Table S1. Literature search strategies

PubMed

#	Searches	Results
**#1**	((((((((((((((Human Papillomavirus Viruses[Mesh]) OR (Human Papillomavirus Viruses[Title/Abstract])) OR (Human Papillomavirus Virus[Title/Abstract])) OR (Papillomavirus Virus, Human[Title/Abstract])) OR (Virus, Human Papillomavirus[Title/Abstract])) OR (Human Papillomavirus[Title/Abstract])) OR (Human Papillomaviruses[Title/Abstract])) OR (HPV, Human Papillomavirus Viruses[Title/Abstract])) OR (Human Papilloma Virus[Title/Abstract])) OR (Human Papilloma Viruses[Title/Abstract])) OR (Papilloma Virus, Human[Title/Abstract])) OR (Virus, Human Papilloma[Title/Abstract])) OR (HPV Human Papillomavirus[Title/Abstract])) OR (HPV Human Papillomaviruses[Title/Abstract])) OR (Human Papillomavirus, HPV[Title/Abstract])) OR (Human Papillomaviruses, HPV[Title/Abstract])	**51149**
**#2**	(((Male [Mesh])) OR (Male [Title/Abstract])) OR (Males [Title/Abstract])	**7,620358**
**#3**	(((((Vaccines [Mesh]) OR (Vaccines [Title/Abstract])) OR (Vaccine [Title/Abstract])) OR (immuni*[Title/Abstract])) OR (jab [Title/Abstract])) OR (inoculat*[Title/Abstract])	**695156**
**#4**	(((((hesitance*[Title/Abstract]) OR (willing*[Title/Abstract])) OR (accept*[Title/Abstract])) OR (perception [Title/Abstract])) OR (attitude [Title/Abstract])) OR (recognition[Title/Abstract])	**1173256**
**#5**	**#1 and #2 and #3 and #4**	**651**

Web of Science

#	Searches	Results
**#1**	TS= (Human Papillomavirus Viruses OR Human Papillomavirus Viruses OR Human Papillomavirus Virus OR Papillomavirus Virus, Human OR Virus, Human Papillomavirus OR Human Papillomavirus OR Human Papillomaviruses OR HPV, Human Papillomavirus Viruses OR Human Papilloma Virus OR Human Papilloma Viruses OR Papilloma Virus, Human OR Virus, Human Papilloma OR HPV Human Papillomavirus OR HPV Human Papillomaviruses OR Human Papillomavirus, HPV OR Human Papillomaviruses, HPV)	**65586**
**#2**	TS= (Male OR Male OR Males)	**1402918**
**#3**	TS= (Vaccines OR Vaccines OR Vaccine OR immuni* OR jab OR inoculat*)	**846903**
**#4**	TS= (hesitance* OR willing* OR accept* OR perception OR attitude OR recognition)	**2992423**
**#5**	**#1 and #2 and #3 and #4**	**475**

EMBASE

#	Searches	Results
**#1**	'papillomavirus vaccines': ab, ti OR 'human papillomavirus vaccines': ab, ti OR 'human papillomavirus virus': ab, ti OR 'papillomavirus virus, human': ab, ti OR 'virus, human papillomavirus': ab, ti OR 'human papillomavirus': ab, ti OR 'human papillomaviruses': ab, ti OR 'hpv, human papillomavirus viruses': ab, ti OR ' human papilloma virus': ab, ti OR 'human papilloma viruses': ab, ti OR 'papilloma virus, human': ab, ti OR 'virus, human papilloma': ab, ti OR 'hpv human papillomavirus': ab, ti OR 'hpv human papillomaviruses': ab, ti OR 'human papillomavirus, hpv': ab, ti OR 'human papillomaviruses, hpv': ab, ti	**60352**
**#2**	male: ab, ti OR males: ab, ti	**2166904**
**#3**	vaccines: ab, ti OR vaccine: ab, ti OR immuni*: ab, ti OR jab: ab ti OR inoculat*: ab, ti	**842051**
**#4**	hesitance*: ab, ti OR willing*: ab, ti OR accept*: ab, ti OR perception: ab, ti OR attitude: ab, ti OR recognition: ab, ti	**1552513**
**#5**	**#1 and #2 and #3 and #4**	**305**

Ovid

#	Searches	Results
**#1**	(Papillomavirus Vaccines or Human Papillomavirus Vaccines or Human Papillomavirus Virus or Papillomavirus Virus, Human or Virus, Human Papillomavirus or Human Papillomavirus or Human Papillomaviruses or HPV, Human Papillomavirus Viruses or Human Papilloma Virus or Human Papilloma Viruses or Papilloma Virus, Human or Virus, Human Papilloma or HPV Human Papillomavirus or HPV Human Papillomaviruses or Human Papillomavirus, HPV or Human Papillomaviruses, HPV).mp. [mp=title, abstract, full text, caption text]	**10265**
**#2**	(Male or Males).mp. [mp=title, abstract, full text, caption text]	**385553**
**#3**	(Vaccines or Vaccine or immuni* or jab or inoculat*).mp. [mp=title, abstract, full text, caption text]	**88303**
**#4**	(hesitance* or willing* or accept* or perception or attitude or recognition).mp. [mp=title, abstract, full text, caption text]	**885986**
**#5**	**#1 and #2 and #3 and #4**	**914**

CNKI

#	Searches	Results
**#1**	（主题：HPV（精确））OR（主题：人乳头状瘤病毒（精确））OR（主题：人乳头状病毒（精确））OR（主题：人乳头瘤病毒（精确））OR（主题：	**56**

#	Searches	Results
	Human Papillomavirus (精确) AND（(主题：HPV 疫苗（精确））OR（主题：疫苗（精确）OR（主题：宫颈癌疫苗（精确））OR（主题：子宫颈癌疫苗（精确））OR（主题：二价（精确））OR（主题：四价（精确））OR（主题：九价（精确））AND（(主题：男性（精确））OR（主题：男（精确））OR（主题：男孩（精确））OR（主题：MSM（精确））AND（(摘要：犹豫（精确））OR（摘要：知识（精确））OR（摘要：安全性（精确））OR（摘要：知晓（精确））OR（摘要：意愿（精确））OR（摘要：接受（精确））OR（摘要：认知（精确））OR（摘要：影响因素（精确））OR（摘要：现状（精确））OR（摘要：安全性（精确）））	

CNKI (China National Knowledge Infrastructure) is a Chinese literature search database. The English translation of the above table is as follows:

CNKI

#	Searches	Results
**#1**	(MeSH Terms： HPV(precise)) OR (MeSH Terms: human papillomavirus (precise)) OR (MeSH Terms: human papillomavirus (precise)) OR (MeSH Terms: human papillomavirus (precise)) OR (MeSH Terms: Human Papillomavirus (precise) AND ((MeSH Terms: HPV vaccine (precise)) OR (MeSH Terms: vaccine (precise) OR (MeSH Terms : cervical cancer vaccine (precise)) OR (MeSH Terms: cervical cancer vaccine (precise)) OR (MeSH Terms: bivalent (precise)) OR (MeSH Terms: quadrivalent (precise)) OR (MeSH Terms: 9-valent (precise)) AND ((MeSH Terms: male (precise)) OR (MeSH Terms: men (precise)) OR (MeSH Terms: boy (precise)) OR (MeSH Terms: MSM (precise)) AND ((abstract: hesitancy (precise)) OR (abstract : knowledge (precise)) OR (abstract: safety (precise)) OR (abstract: awareness (precise)) OR (abstract: willingness (precise)) OR (abstract: acceptance (precise)) OR (abstract: cognitively (precise)) OR(abstract: Influencing factors (precise)) OR (abstract: status (precise)) OR (abstract : safety (precise)))	**56**

Wan Fang

#	Searches	Results
**#1**	主题：(HPV or 人乳头状瘤病毒 or 人乳头状病毒 or 人乳头瘤病毒 or Human Papillomavirus) and 主题：(HPV 疫苗 or 疫苗 or 宫颈癌疫苗 or 子宫颈癌疫苗 or 二价 or 四价 or 九价） and 主题：(男性 or 男 or 男孩 or MSM) and 主题：(犹豫 or 知识 or 知晓 or 意愿 or 接受 or 认知 or 影响因素 or 现状 or 安全性)	**88**

Wan Fang (Wan Fang Data) is a Chinese literature search database. The English translation of the above table is as follows:

Wan Fang

#	Searches	Results
**#1**	MeSH Terms:(HPV or Human papillomavirus or Human papillomavirus or Human papillomavirus or Human Papillomavirus) and MeSH Terms:(HPV vaccine or vaccine or cervical cancer vaccine or Cervical cancer vaccine or bivalent or quadrivalent or9-valent) and MeSH Terms: (male or men or boy or MSM) and MeSH Terms: (hesitancy or knowledge or awareness or willingness or acceptance or cognitively or Influencing factors or status or safety)	**88**

Table S2. Agency for Healthcare Research and Quality (AHRQ) Scale assessment items and scoring criteria

Item	Yes	No	Unclear
1) Define the source of information (survey, record review)			
2) List inclusion and exclusion criteria for exposed and unexposed subjects (cases and controls) or refer to previous publications			
3) Indicate time period used for identifying patients			
4) Indicate whether or not subjects were consecutive if not population-based			
5) Indicate if evaluators of subjective components of study were masked to other aspects of the status of the participants			
6) Describe any assessments undertaken for quality assurance purposes (e.g., test/retest of primary outcome measurements)			
7) Explain any patient exclusions from analysis			
Item	Yes	No	Unclear
8) Describe how confounding was assessed and/or controlled.			
9) If applicable, explain how missing data were handled in the analysis			
10) Summarize patient response rates and completeness of data collection			

11) Clarify what follow-up, if any, was expected and the percentage of patients for which incomplete data or follow-up was obtained			

Scoring criteria: each entry is evaluated using "yes," "no," and "unclear," with "yes" counting as 1 point and "no" or "unclear" counting as 0 points, and the scores of each entry are added up to the total score (0~11 points). A score of 0 to 3 was set as low quality, 4 to 7 as medium quality, and 8 to 11 as high quality.

Table S3. Literature quality assessment

Studies	Item1	Item2	Item3	Item4	Item5	Item6	Item7	Item8	Item9	Item10	Item11	quality assessment
Ferris, D., et al.	yes	yes	yes	yes	yes	yes	yes	yes	no	yes	unclear	High
Ferris, D., et al.	yes	yes	yes	yes	yes	yes	yes	yes	no	yes	unclear	High
Gottvall, M., et al.	yes	no	yes	yes	no	yes	yes	yes	yes	yes	unclear	High
Daley, E., et al.	yes	yes	yes	yes	yes	no	yes	yes	no	yes	unclear	High
Hernandez, B. Y., et al.	yes	yes	yes	yes	no	no	yes	no	no	yes	unclear	Moderate
Reiter, P., et al.	yes	yes	yes	yes	no	yes	yes	yes	no	yes	unclear	High
Reiter, P. L., et al.	yes	no	yes	yes	yes	yes	yes	yes	yes	yes	unclear	High
Oh, J. K., et al.	yes	yes	yes	yes	no	no	yes	yes	yes	yes	unclear	High
Gilbert, P., et al.	yes	no	yes	yes	yes	no	yes	yes	yes	yes	unclear	High
Studies	Item1	Item2	Item3	Item4	Item5	Item6	Item7	Item8	Item9	Item10	Item11	quality assessment
Gilbert, P., et al.	yes	no	yes	yes	yes	yes	yes	yes	no	yes	unclear	High
Katz, M. L., et al.	yes	no	yes	yes	no	unclear	no	no	no	yes	unclear	Moderate
Wheldon, C. W., et al.	yes	yes	yes	yes	no	no	yes	yes	yes	yes	unclear	High
Thomas, Emily A., et al.	yes	yes	yes	yes	unclear	no	no	yes	no	yes	unclear	Moderate

Richard A., et al.	yes	yes	yes	yes	no	no	no	yes	yes	yes	unclear	Moderate
Colon-Lopez, V., et al.	yes	yes	yes	yes	no	no	yes	no	yes	yes	unclear	Moderate
Forster, A. S., et al.	yes	yes	yes	yes	yes	no	yes	yes	no	yes	unclear	High
Rank, C., et al.	yes	yes	yes	yes	yes	no	yes	yes	no	yes	unclear	High
Al-Naggar RA.	yes	yes	yes	yes	unclear	no	unclear	yes	no	yes	unclear	Moderate
Blodt, S., et al.	yes	yes	yes	yes	no	no	yes	yes	no	yes	unclear	Moderate
Gutierrez Jr, B., et al.	yes	yes	yes	yes	no	yes	no	yes	no	yes	unclear	Moderate
Gray, Clive M., et al.	yes	yes	yes	yes	unclear	no	yes	yes	yes	yes	unclear	High
Belani, H. K., et al.	yes	yes	yes	yes	no	unclear	yes	no	yes	yes	unclear	Moderate
Gellenoncourt, A. and P. Di Patrizio	yes	yes	yes	yes	yes	unclear	yes	yes	yes	yes	unclear	High
Zou, H. C., et al.	yes	yes	yes	yes	no	no	unclear	yes	yes	yes	unclear	Moderate
Khurana, S., et al.	yes	yes	yes	yes	unclear	no	yes	yes	yes	yes	unclear	High
Giuliani, M., et al.	yes	yes	yes	yes	yes	no	yes	yes	no	yes	unclear	High
Hunter, T. and M. Weinstein	yes	yes	yes	yes	no	no	unclear	yes	yes	unclear	unclear	Moderate
Napolitano, F., et al.	yes	yes	yes	yes	yes	yes	no	yes	no	yes	unclear	High
Zou, H. C., et al.	yes	yes	yes	yes	no	no	yes	yes	no	yes	unclear	Moderate

Studies	Item1	Item2	Item3	Item4	Item5	Item6	Item7	Item8	Item9	Item10	Item11	quality assessment
Zhu,J.H., et al.	yes	no	yes	yes	no	no	no	yes	yes	yes	unclear	Moderate
C. Sadlier, et al.	yes	yes	yes	yes	unclear	no	no	yes	no	yes	unclear	Moderate
Stephens, Dionne P., et al.	yes	yes	no	yes	unclear	no	no	yes	yes	yes	unclear	Moderate
Tian, T.	yes	yes	yes	yes	no	no	yes	yes	yes	yes	unclear	High

Hoefer, Lea, et al.	yes	yes	yes	yes	no	no	yes	yes	no	yes	unclear	Moderate
Cheung, T., et al.	yes	yes	yes	yes	yes	no	yes	yes	no	yes	unclear	High
Stanley, C., et al.	yes	yes	yes	yes	no	yes	yes	yes	yes	yes	unclear	High
Cooper, Dexter L., et al.	yes	no	yes	yes	no	no	unclear	yes	no	yes	unclear	Moderate
Nadarzynski, T., et al.	yes	yes	yes	yes	unclear	no	yes	yes	no	yes	unclear	Moderate
Tian, T., et al.	yes	yes	yes	yes	no	yes	no	yes	unclear	yes	unclear	Moderate
Wong, L. P., et al.	yes	yes	yes	yes	no	yes	yes	yes	no	yes	unclear	High
Kuang.J.Y., et al.	no	yes	yes	yes	no	no	no	unclear	unclear	unclear	unclear	low
li.Y.F., et al.	yes	yes	yes	yes	yes	yes	no	yes	yes	yes	unclear	High
Wang, S., et al.	yes	yes	yes	yes	yes	yes	unclear	yes	yes	yes	unclear	High
Ren.W.J., et al.	yes	yes	no	yes	unclear	yes	no	yes	yes	yes	unclear	Moderate
Farsi, N. J., et al.	yes	yes	yes	yes	yes	no	yes	yes	no	yes	unclear	High
Bai.M.M.	yes	yes	yes	yes	unclear	yes	yes	yes	no	yes	unclear	High
Wei He, et al.	yes	yes	yes	yes	no	no	yes	yes	yes	yes	unclear	High
Ye, Ze-Hao, et al.	yes	yes	yes	yes	yes	no	no	yes	no	yes	unclear	Moderate
Chen, Q., et al.	yes	no	no	yes	unclear	no	yes	yes	yes	yes	unclear	Moderate

Studies	Item1	Item2	Item3	Item4	Item5	Item6	Item7	Item8	Item9	Item10	Item11	quality assessment
Pan, H., et al.	yes	yes	yes	yes	unclear	no	yes	yes	yes	yes	unclear	High
Pham, D., et al.	yes	yes	yes	yes	unclear	no	yes	yes	no	yes	unclear	Moderate
Ran, H., et al.	yes	yes	no	yes	yes	yes	yes	yes	no	yes	unclear	High
Shi.J., et al.	yes	yes	yes	yes	unclear	no	no	yes	yes	yes	unclear	Moderate
Zhang.H., et al.	yes	yes	yes	yes	no	yes	no	yes	yes	yes	unclear	High

Zhang.J., et al.	yes	yes	yes	yes	no	no	yes	no	no	yes	unclear	Moderate
Gursoy, M. Y. and F. Sagtas	yes	yes	yes	yes	unclear	no	unclear	yes	unclear	yes	unclear	Moderate
Lv.H.W, et al.	yes	yes	yes	yes	no	yes	yes	no	unclear	yes	unclear	Moderate

Table S4. HPV vaccine acceptance in different WHO areas and countries

Countries and regions	no. of studies	sample size	acceptance rate (%)	95%-CI
**Americas region of WHO**	19	8555	57.12	[0.4592; 0.6798]
United States	17	7472	55.12	[0.4300; 0.6694]
Canada	2	1083	72.43	[0.5890; 0.8417]
**Eastern Mediterranean Region of WHO**	2	873	47.54	[0.4423; 0.5086]
U.A.E	1	356	46.07	[0.4091; 0.5127]
Saudi Arabia	1	517	48.55	[0.4425; 0.5286]
**European Region of WHO**	10	6028	48.09	[0.2963; 0.6682]
UK	2	1823	63.48	[0.2130; 0.9581]
Italy	2	1159	64.59	[0.5121; 0.7690]
France	1	328	34.45	[0.2940; 0.3969]
Turkey	1	1646	10.45	[0.0902; 0.1198]
Ireland	1	281	30.96	[0.2568; 0.3650]
Greece	1	298	76.85	[0.7188; 0.8147]
Sweden	1	261	74.33	[0.6884; 0.7946]
Germany	1	232	8.19	[0.0497; 0.1210]
**South-East Asia Region of WHO**	1	178	89.89	[0.8499; 0.9393]
India	1	178	89.89	[0.8499; 0.9393]
**Western Pacific Region of WHO**	24	17328	50.81	[0.4073; 0.6087]
China	20	13459	51.52	[0.3959; 0.6337]
Malaysia	2	3173	51.46	[0.4462; 0.5828]
Australia	1	200	30.00	[0.2383; 0.3655]
Korea	1	496	55.44	[0.5105; 0.5980]

WHO, World Health Organization
